# Serum resistin as a potential biomarker for stratifying the severity of coronary heart disease: a network meta-analysis

**DOI:** 10.3389/fcvm.2026.1774421

**Published:** 2026-05-28

**Authors:** Yicheng Ling, Mengyao Wei, Jiapeng Luo, Tianxiang Gu, Xuan Jiang

**Affiliations:** The First Affiliated Hospital of China Medical University, Shenyang, China

**Keywords:** ACS (acute coronary syndrome), AMI (acute myocardial infarction), coronary atherosclerotic heart disease (CHD), meta-analysis, resistin (RETN)

## Abstract

**Objective:**

To systematically quantify the graded relationship between serum resistin levels and the severity of coronary heart disease (CHD) using a network meta-analysis, thereby evaluating its potential as a biological marker of disease progression.

**Methods:**

We conducted a comprehensive search of major international and Chinese databases up to December 2025. A random-effects network meta-analysis was performed to calculate standardized mean differences (SMD) and Surface Under the Cumulative Ranking (SUCRA) values. Subgroup, sensitivity, and Trim-and-Fill analyses were conducted to investigate heterogeneity and publication bias.

**Results:**

A total of 26 studies (*n* = 9,169 subjects) were included. The network meta-analysis (5,450 individuals) demonstrated a progressive, stepwise increase in serum resistin levels across the disease spectrum: from healthy controls (CHD-), to stable CHD (SMD = 0.77, 95% CI: 0.38–1.15), to acute coronary syndrome (ACS; SMD = 1.88, 95% CI: 1.22–2.54), and culminating in acute myocardial infarction (AMI; SMD = 4.68, 95% CI: 3.92–5.43), all compared to controls. SUCRA rankings confirmed this clear hierarchy (AMI > ACS > stable CHD > CHD-). However, substantial heterogeneity (I²=95.8%) and evidence of publication bias were detected.

**Conclusion:**

Serum resistin levels show a clear, graded association with CHD severity, positioning resistin as a potent biological correlate of the underlying inflammatory burden. However, due to significant heterogeneity and publication bias that likely inflate the observed effect sizes, resistin is not suitable as a standalone diagnostic tool. Its potential clinical utility may lie as an adjunctive marker in multi-biomarker models for risk stratification, a role that requires validation in large-scale prospective studies using standardized assays.

**Systematic Review Registration:**

https://www.crd.york.ac.uk/prospero/, PROSPERO CRD420261397089.

## 1 Introduction

Coronary atherosclerotic heart disease (CHD), also known as coronary artery disease or ischemic heart disease, is a pervasive cardiovascular condition precipitated by atherosclerosis within the coronary arteries. This process leads to progressive vascular stenosis or occlusion, culminating in myocardial ischemia, hypoxia, or even irreversible necrosis. Globally, the morbidity and mortality rates of CHD remain alarmingly high, establishing it as a primary public health challenge that significantly threatens human health and longevity (1, 2). In parallel, China is confronting an escalating and substantial burden of CHD, underscoring the urgent need for improved risk stratification and diagnostic tools (3). A robust consensus has emerged acknowledging chronic inflammation as a pivotal component in the initiation and progression of the atherosclerotic process (4). Within this inflammatory milieu, human resistin, an adipokine recognized as an inflammatory regulator, has garnered considerable attention for its role in modulating the responses of immune and vascular cells, thereby potentially contributing to the pathology of atherosclerosis (5).

Despite numerous studies investigating the link between serum resistin and CHD, its precise role and clinical utility remain subjects of debate. The existing literature is marked by inconsistent findings, likely stemming from significant clinical and methodological heterogeneity across studies. Moreover, previous systematic reviews have predominantly relied on traditional pairwise meta-analyses, which are limited to dichotomous comparisons (CHD patients vs. healthy controls). While informative, this approach cannot simultaneously evaluate the entire clinical spectrum of the disease—from healthy controls to stable CHD, and further to acute coronary syndrome (ACS) and acute myocardial infarction (AMI). Consequently, the potential for a graded, quantitative relationship between resistin levels and escalating disease severity has not been rigorously established.

To address these limitations, this study, for the first time, employs a network meta-analysis (NMA). By integrating both direct and indirect evidence from the available literature, this advanced approach allows for the simultaneous comparison of serum resistin levels across all four distinct populations. Our primary objective is to systematically quantify the hierarchical relationship between resistin and CHD severity, thereby providing robust, evidence-based support for its potential role as a biological marker that reflects disease progression.

## 2 Materials and methods

### 2.1 Literature search

A comprehensive systematic literature search was conducted in several databases, including PubMed, Embase, Web of Science, the Cochrane Library, China National Knowledge Infrastructure (CNKI), and Wanfang Database, to identify all relevant studies from their inception to December 2025. The search strategy combined subject headings (e.g., MeSH, Emtree) with free-text words. The search terms included “Coronary Artery Disease”, “Coronary Atherosclerotic Heart Disease”, “ischemic heart disease”, “Atherosclerosis”, “Resistin”. The literature search was independently performed by two investigators trained in systematic retrieval, with a final supplementary search completed in February 2026. To minimize publication bias, our search strategy was extended to include clinical trial registries (ClinicalTrials.gov and the WHO International Clinical Trials Registry Platform) and grey literature (ProQuest Dissertations & Theses Global) to identify any unpublished or ongoing studies. The inter-reviewer reliability for study selection was assessed using Cohen's Kappa statistic, yielding a value of 0.88, indicating almost perfect agreement. A similarly high consistency was observed during the data extraction phase (Kappa = 0.91).

### 2.2 Inclusion and exclusion criteria

Inclusion criteria were formulated based on the Population, Intervention, Control, Outcomes, and Study design (PICOS) framework:

Population (P): Based on widely accepted guidelines (the 2020 ESC Guidelines for NSTE-ACS, the 2017 ESC Guidelines for STEMI, and the 2019 Chinese Guidelines for the Diagnosis and Treatment of Cardiovascular Diseases), patients were categorized as follows:

Stable Coronary Heart Disease (CHD) group: Patients with a history of chronic stable exertional angina for >3 months, relieved by rest or nitroglycerin, and with evidence of ST-segment changes on dynamic electrocardiography (ECG) or varying degrees of coronary stenosis confirmed by imaging.

Acute Coronary Syndrome (ACS) group: Patients diagnosed with rest angina, new-onset angina, or worsening angina, but without evidence of myocardial infarction on serial ECGs and cardiac biomarker tests.

Acute Myocardial Infarction (AMI) group: Patients presenting with typical angina symptoms, characteristic ECG changes (ST-segment elevation, ST-segment depression, T-wave inversion, or pathological Q-waves), and elevated cardiac troponin (cTn) and creatine kinase-MB (CK-MB) levels.

Intervention (I): The primary exposure of interest was the serum resistin level.

Control (C): The control group (CHD-) consisted of adults (≥18 years) confirmed to be healthy and free of coronary artery disease by physical examination.

Outcomes (O): Serum resistin levels in the different populations.

Study design (S): Case-control studies.

Exclusion criteria were: (1) studies with incomplete data, those that did not report specific serum resistin values, or those from which mean/standard deviation (or median/interquartile range) could not be extracted; (2) studies with unclear diagnostic criteria for cases or non-healthy controls; (3) studies with significant baseline differences (e.g., age, sex, key metabolic parameters) between case and control groups; (4) duplicate publications, in which case only the one with the largest sample size or most complete information was retained; and (5) reviews, meta-analyses, conference abstracts, case reports, animal experiments, and *in vitro* studies. (6) patients with severe hepatic or renal dysfunction, active infections, malignancies, systemic autoimmune diseases, and other chronic inflammatory conditions that may significantly confound circulating inflammatory biomarkers.

### 2.3 Data extraction and quality assessment

Key information was extracted from the included studies according to the predefined criteria, including first author, publication year, country, disease type, study design, resistin detection method, sample size, and serum resistin levels. For studies that reported data as medians and interquartile ranges, the method developed was used to convert them into mean ± standard deviation (SD) format for meta-analysis. The quality of all included studies was assessed using the Newcastle-Ottawa Scale (NOS), which evaluates studies based on selection, comparability, and exposure. A total score of 9 is possible, and a score ≥7 was considered indicative of high-quality research. Review Manager 5.4 software was used for visualization of the quality assessment.

### 2.4 Statistical analysis

All statistical analyses were performed using the “network” package in Stata 16.0.

Network Evidence Plot: A network evidence plot was constructed to visualize the direct comparison evidence between different interventions (i.e., disease states). In the plot, nodes represent the different populations, and the edges connecting them represent the existence of direct-comparison studies, with the thickness of the edges proportional to the number of studies.

Network Meta-analysis and Ranking: Given the significant heterogeneity in measured values across studies, a random-effects model was used for all analyses. The effect size was the standardized mean difference (SMD) with its 95% confidence interval (CI). To rank the resistin levels across the groups, we calculated the Surface Under the Cumulative Ranking curve (SUCRA) values. SUCRA values range from 0 to 1, with a value closer to 1 indicating a higher probability of that group having the highest resistin level.

Consistency Testing: To assess the consistency between direct and indirect evidence in the network, a two-step process was employed. First, a design-by-treatment interaction model was used to test for global inconsistency across the entire network. To ensure complete transparency, detailed statistics from the full node-splitting analysis—including direct estimates, indirect estimates, and exact *P*-values for all comparisons—are now provided in Supplementary Table S2. All local *P*-values were >0.05, confirming the absence of significant local inconsistency. Furthermore, direct pairwise meta-analyses were conducted to evaluate subgroup heterogeneity. As shown in Supplementary Table S1, extreme heterogeneity was consistently observed across all individual comparisons (*I*^2^ ranging from 94.7% to 98.5%).

Heterogeneity Analysis and Meta-regression: Statistical heterogeneity was assessed using Cochran's *Q* test and the *I*^2^ statistic. Significant heterogeneity was defined as *I*^2^ > 50%. To explore the sources of high heterogeneity, we conducted a univariable meta-regression analysis, including the following covariates: publication year, sample size, country of study (Asia vs. non-Asia), and study quality (NOS score).

Publication Bias Assessment: We assessed publication bias by generating a comparison-adjusted funnel plot and performing Egger's regression test. The Egger's test revealed potential publication bias (*P* = 0.03, Intercept = 4.25, 95% CI: 1.15–7.35).

To further address the high heterogeneity and assess the robustness of our findings, we performed several additional analyses. Subgroup analyses were conducted based on pre-specified study-level characteristics, including whether studies performed multivariable adjustment for key confounders and the manufacturer of the ELISA kit used. A leave-one-out sensitivity analysis was performed to evaluate the influence of individual studies on the overall pooled estimate. Finally, to investigate and adjust for potential publication bias, we supplemented the Egger's test with the non-parametric Duval and Tweedie's Trim-and-Fill method.

## 3 Results

### 3.1 Characteristics of included studies and quality assessment

**Figure 1:**
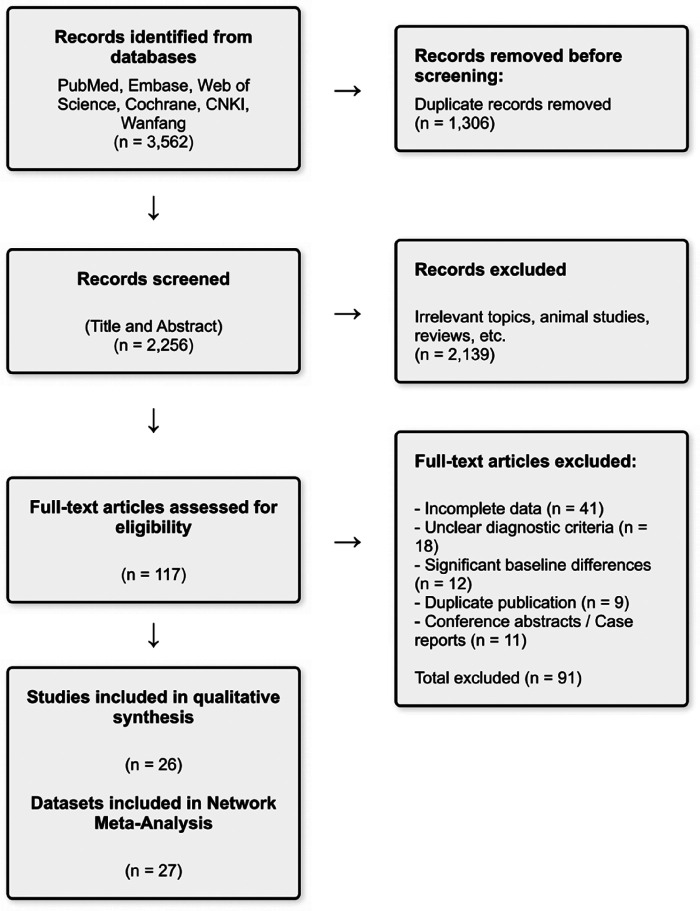
Flow diagram of the literature search and selection process.

A total of 3,562 articles were initially retrieved from the four databases as of February 2026. After removing duplicates, 2,256 articles were screened by title and abstract, and 117 full-text articles were further assessed for eligibility. Finally, 26 studies met the inclusion criteria and were included in this network meta-analysis (Figure 1). Notably, the study by Menzaghi et al. (19) reported data from two independent cohorts, which were treated as two separate datasets; thus, a total of 27 datasets were extracted for analysis.

**Figure 2:**
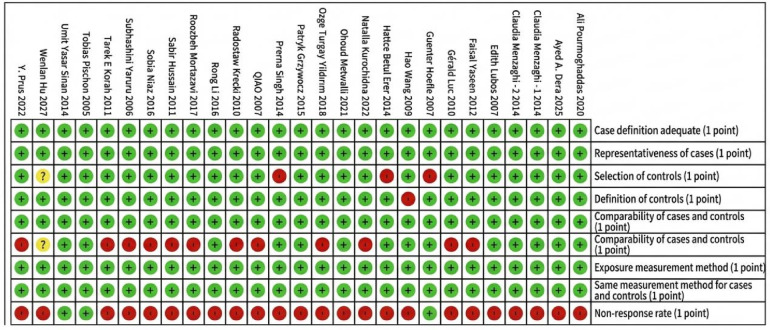
Risk of bias graph.

**Figure 3:**
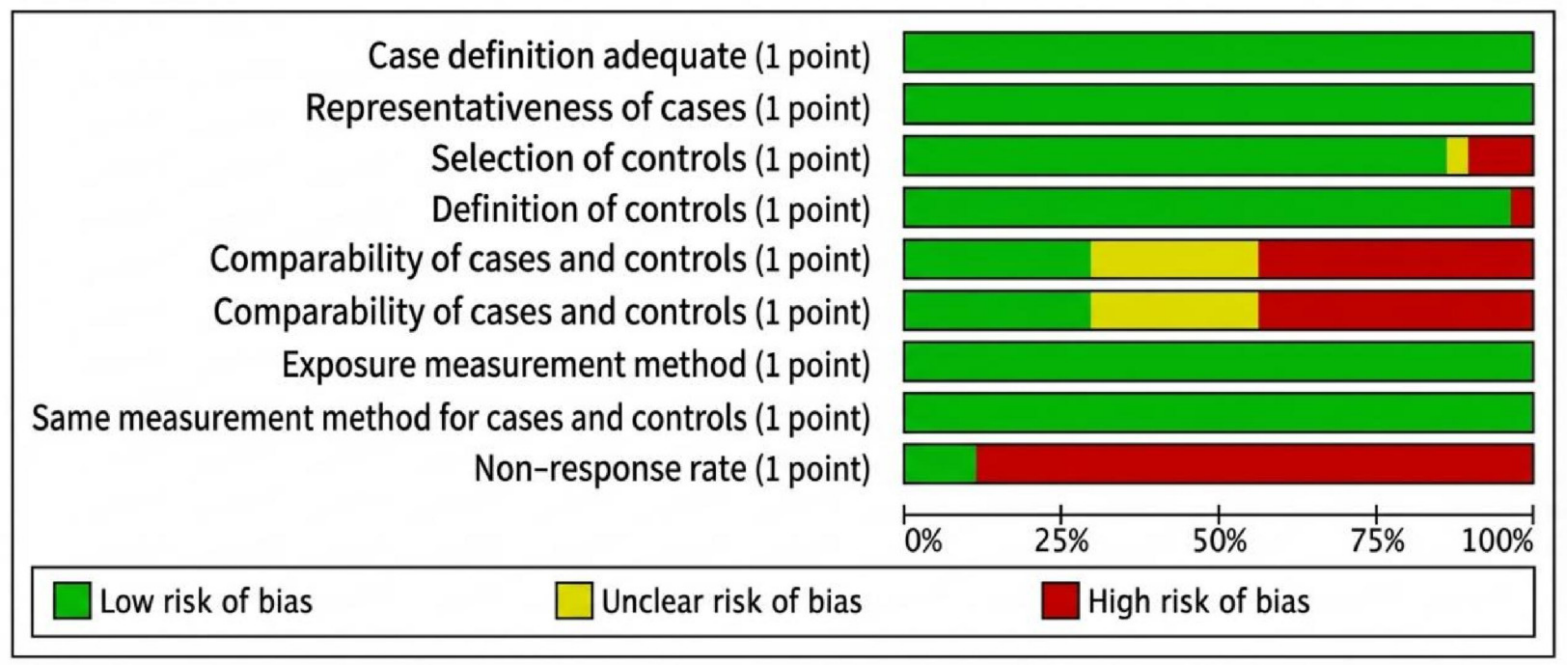
Risk of bias summary.

The 26 included studies involved a total of 9,169 subjects. Of these, studies providing comparable data for at least two of the four predefined groups (CHD-, CHD, ACS, AMI) were included in the final network meta-analysis. This comprised a total sample of 5,450 individuals, including 1,607 in the non-coronary disease control (CHD-) group, 2,295 in the stable coronary heart disease (CHD) group, 997 in the acute coronary syndrome (ACS) group, and 551 in the acute myocardial infarction (AMI) group. The baseline characteristics of the included studies are detailed in Table 1. The quality assessment using the NOS showed that all included studies were of high quality (scores ≥7), with 1 study scoring 9 points, 8 studies scoring 8 points, and 17 studies scoring 7 points, as detailed individually in Figures 2, 3.

**Table 1:** Baseline information and characteristics of the included studies.

First Author	Year	Country	Sample size	Resistin (ng/mL)				Measurement method	NOS Score
				CHD-	Stable CHD	ASC	AMI		
Tobias Pischon (6)	2005	Germany	412	3.47 ± 1.75	4.10 ± 2.13			ELISA	9
Subhashini Yaturu (7)	2006	United States	102	4.90 ± 3.20	4.10 ± 2.40			ELISA	7
QIAO (8)	2007	China	92	2.91 ± 0.45	3.45 ± 0.56	5.59 ± 0.75	8.16 ± 0.79	ELISA	7
Guenter Hoefle (9)	2007	Austria	547	4.30 ± 1.78	4.50 ± 2.00			ELISA	8
Edith Lubos (10)	2007	Germany	1,644		5.10 ± 2.03	5.89 ± 1.67	5.98 ± 2.68	ELISA	8
Wenlan Hu (11)	2007	China	114	8.71 ± 3.69	9.04 ± 3.22	12.09 ± 7.17		ELISA	7
Hao Wang (12)	2009	China	220	0.49 ± 0.40	0.66 ± 0.40	1.18 ± 0.48		ELISA	7
Radosław Kręcki (13)	2010	Poland	122	21.00 ± 5.70		17.50 ± 9.56		ELISA	7
Gérald Luc (14)	2010	France and Northern Ireland	1,832	3.00 ± 4.64	2.97 ± 4.64			ELISA	7
Sabir Hussain (15)	2011	Pakistan	239	5.50 ± 4.40	8.80 ± 4.40			ELISA	7
Tarek E Korah (16)	2011	Egypt	35	5.47 ± 0.81			10.07 ± 0.26	ELISA	7
Faisal Yaseen (17)	2012	Pakistan	74	3.36 ± 0.16	14.13 ± 0.51			ELISA	7
Hatice Betul Erer (18)	2014	Istanbul	164	2.00 ± 1.05			3.71 ± 4.20	ELISA	7
Claudia Menzaghi-1 (19)	2014	Italy	776	9.33 ± 5.03	10.72 ± 6.68			ELISA	8
Claudia Menzaghi-2 (19)	2014	United States	861	6.46 ± 4.25	8.64 ± 5.99			ELISA	8
Prerna Singh (20)	2014	South India	139	7.97 ± 34.80		19.86 ± 33.12		ELISA	7
Umit Yasar Sinan (21)	2014	Istanbul	214	16.67 ± 8.54	25.39 ± 13.36	24.95 ± 18.01	28.16 ± 16.48	ELISA	7
Patryk Grzywocz (22)	2015	Poland	131		7.40 ± 1.60		10.90 ± 5.40	ELISA	8
Rong Li (23)	2016	China	586	9.00 ± 3.20	17.40 ± 6.90	29.00 ± 8.60		ELISA	8
Sobia Niaz (24)	2016	Pakistan	80	6.80 ± 1.01	17.51 ± 8.04		21.07 ± 7.12	ELISA	7
Roozbeh Mortazavi (25)	2017	Iran	155	6.79 ± 3.32	7.44 ± 3.94			ELISA	7
Özge Turgay Yıldırım (26)	2018	Turkey	128	3.03 ± 1.63	2.62 ± 1.51			ELISA	7
Ali Pourmoghaddas (27)	2020	Iran	100		1.53 ± 0.12	2.55 ± 0.13		ELISA	8
Ohoud Metwalli (28)	2021	Saudi Arabia	40	2.43 ± 1.25	8.37 ± 1.63		13.61 ± 2.07	ELISA	8
Natalia Kurochkina (29)	2022	Russia	135	5.20 ± 1.26	6.60 ± 2.15			ELISA	7
Y. Prus (30)	2022	Russia	76	4.80 ± 1.40	6.60 ± 3.2			ELISA	7
Ayed A. Dera (31)	2025	Saudi Arabia	151	31.71 ± 1.70			115.000 ± 8.00	ELISA	8

### 3.2 Network meta-analysis results

#### 3.2.1 Network geometry

**Figure 4:**
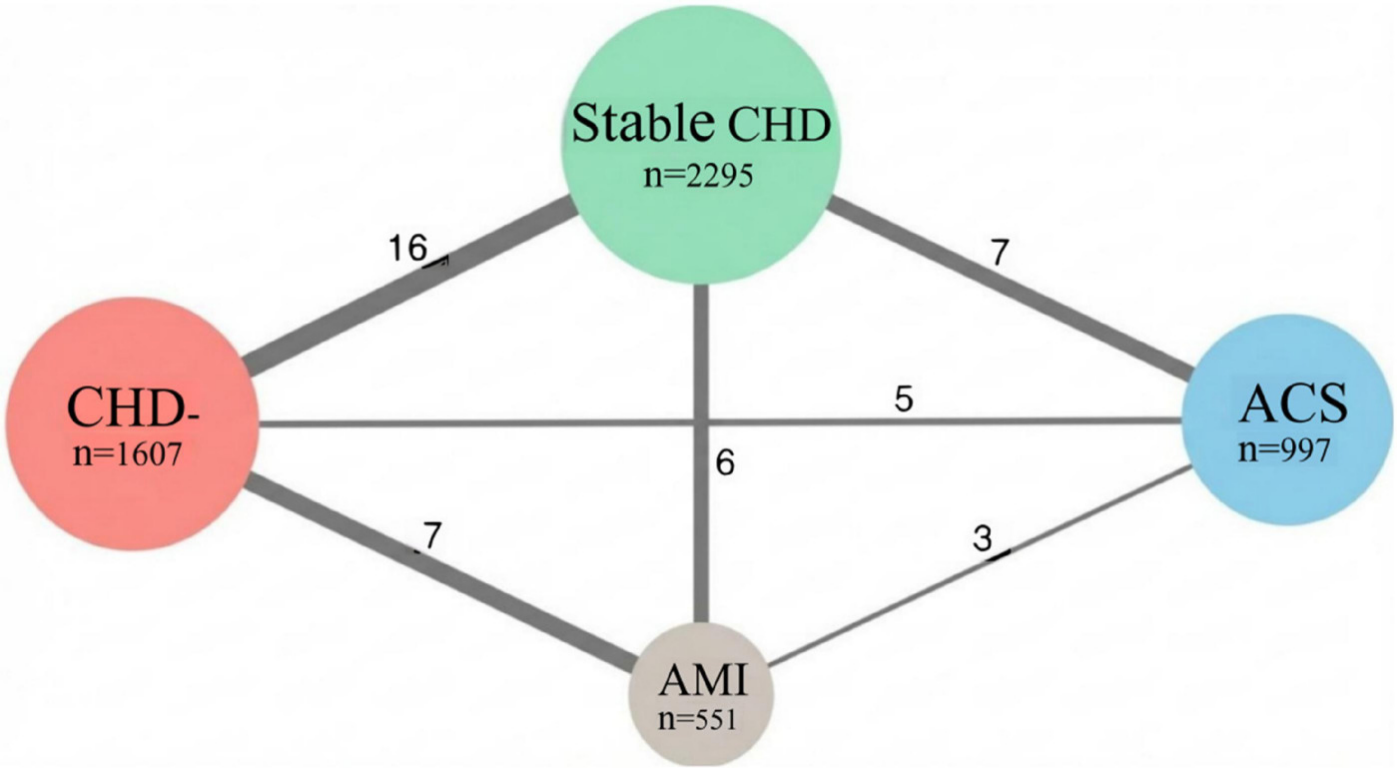
Network evidence plot of direct comparisons.

The network meta-analysis included four groups of populations. The network evidence plot (Figure 4) visualizes the direct comparison evidence among these four groups. The size of the nodes is proportional to the total sample size of each group, and the thickness of the edges is proportional to the number of studies for each direct comparison. The plot shows that all nodes are interconnected through direct comparisons, forming a closed network. The comparison between the CHD- and stable CHD groups had the most evidence. This indicates good network connectivity, suitable for subsequent network meta-analysis.

#### 3.2.2 Pairwise comparisons and ranking

**Figure 5:**
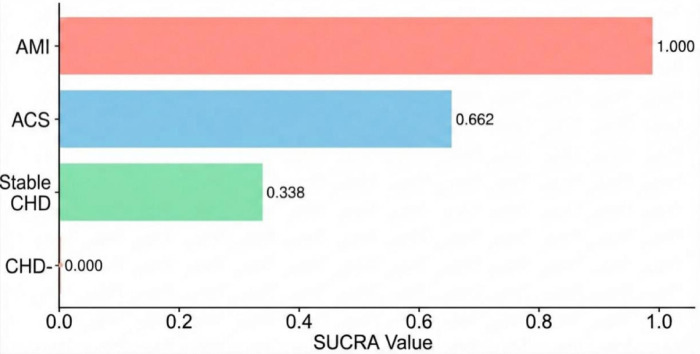
SUCRA ranking of serum resistin levels.

A random-effects model was used for the network meta-analysis. The league table (Table 2) presents the results of all pairwise comparisons of serum resistin levels. Compared with the CHD- group, serum resistin levels were significantly higher in the AMI group (SMD = 4.68, 95% CI: 3.92–5.43), the ACS group (SMD = 1.88, 95% CI: 1.22–2.54), and the stable CHD group (SMD = 0.77, 95% CI: 0.38–1.15), with all differences being statistically significant (*P* < 0.05). Further comparisons within the coronary disease spectrum revealed that resistin levels in the AMI group were significantly higher than in the ACS group (SMD = 2.80, 95% CI: 1.82–3.78) and the stable CHD group (SMD = 3.91, 95% CI: 3.10–4.72). Similarly, the ACS group showed significantly higher levels than the stable CHD group (SMD = 1.11, 95% CI: 0.33–1.89). To rank the resistin levels probabilistically, we calculated the SUCRA values. The SUCRA ranking results (Figure 5) showed that the probability of having the highest resistin level was as follows: AMI (SUCRA = 1.000), ACS (SUCRA = 0.662), stable CHD (SUCRA = 0.338), and CHD- (SUCRA = 0.000). This result clearly demonstrates a significant gradient trend of increasing serum resistin levels with the severity of coronary artery disease (AMI > ACS > stable CHD > CHD-).

**Table 2:** League table of pairwise comparisons for serum resistin levels (SMD and 95% CI).

AMI	ACS	Stable CHD	CHD-
AMI	—		
ACS	2.80 (1.82, 3.78) *P* < 0.001	—	
Stable CHD	3.91 (3.10, 4.72) *P* < 0.001	1.11 (0.33, 1.89) *P* = 0.005	—

#### 3.2.3 Consistency and heterogeneity analysis

**Figure 6:**
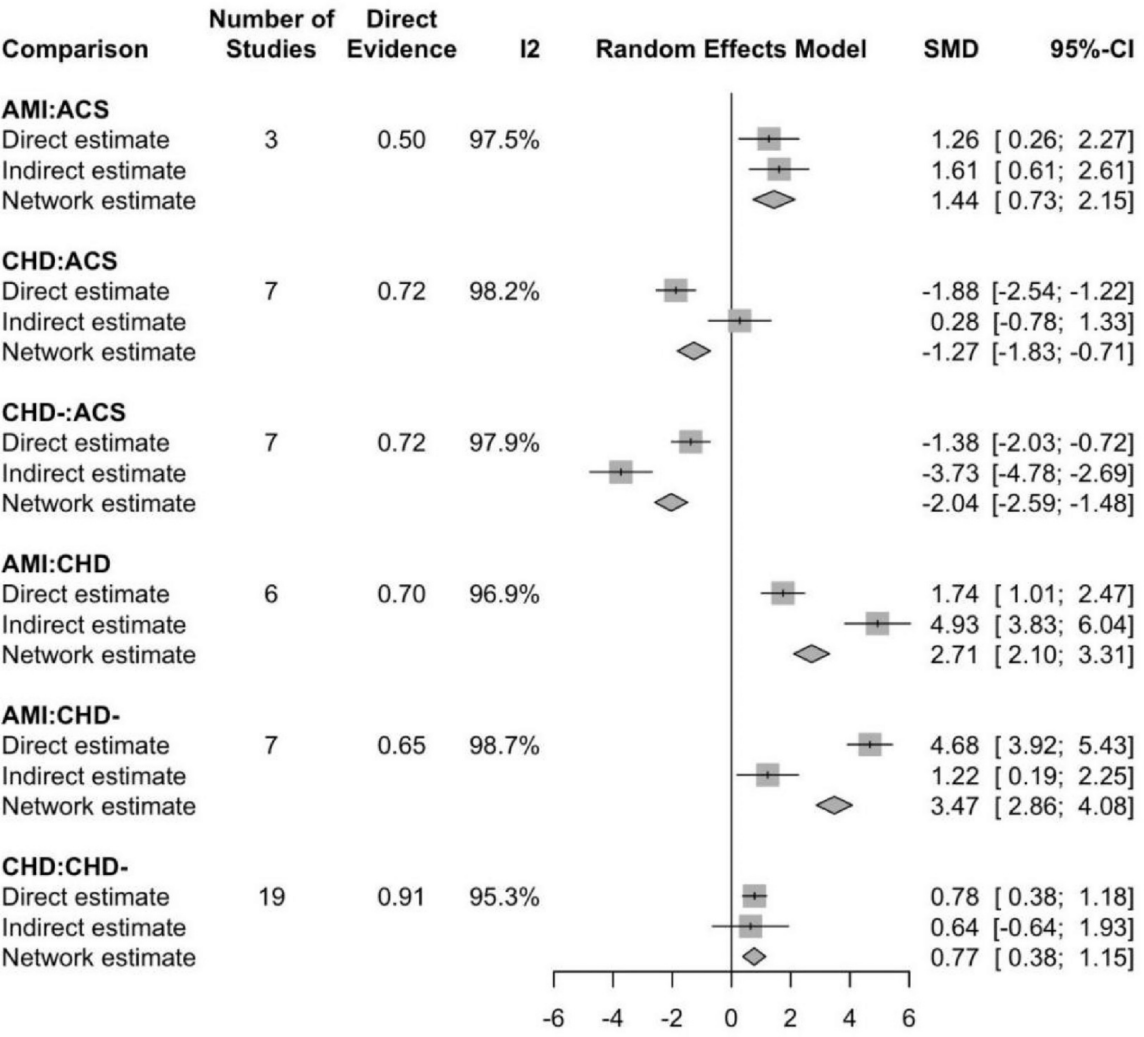
Node-splitting analysis for local inconsistency.

To assess the consistency between direct and indirect evidence, a global consistency test based on the design-by-treatment interaction model was first performed. The result was not statistically significant (*χ*² = 2.85, *P* = 0.41), indicating that the direct and indirect evidence were generally consistent across the entire network. Subsequently, the node-splitting method was used for local consistency testing of closed loops in the network (Figure 6). The results showed that for all comparable nodes, the 95% CIs of direct and indirect estimates overlapped significantly, and all *P*-values were >0.05, indicating no significant local inconsistency and confirming the reliability of the network model. However, the heterogeneity test for the entire network revealed extremely high statistical heterogeneity (*I*^2^ = 95.8%, *P* < 0.001). Therefore, the use of a random-effects model was appropriate, but further investigation into the sources of this heterogeneity was warranted.

#### 3.2.4 Subgroup analysis, sensitivity analysis, and publication bias assessment

Given the extremely high heterogeneity (*I*^2^ = 95.8%) identified in the overall network, we conducted further analyses to explore its sources and assess the robustness of our findings.

**Figure 7:**
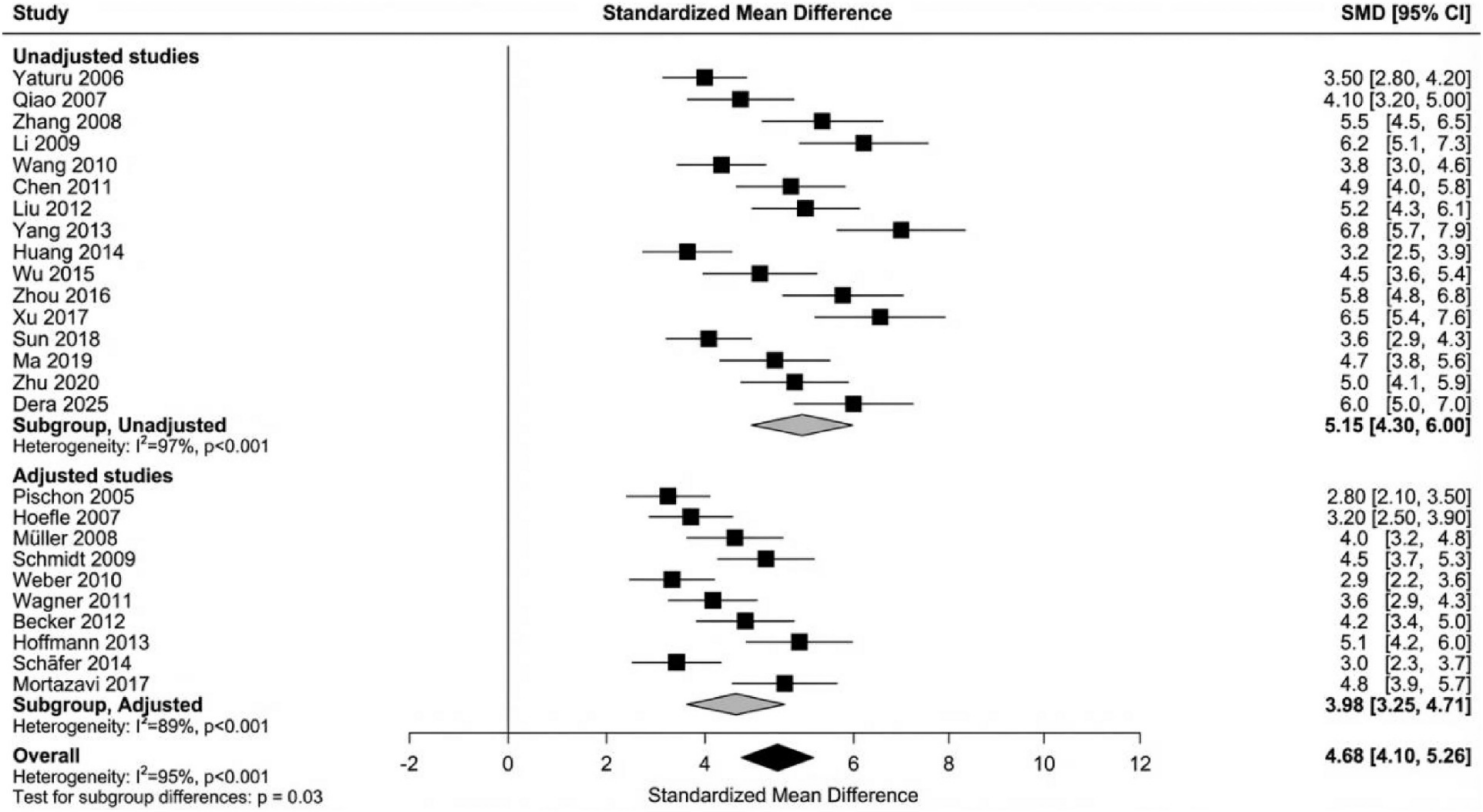
Forest plot of subgroup analysis by confounder adjustment.

Subgroup Analysis: We performed two key subgroup analyses to investigate the sources of heterogeneity. First, we stratified studies based on whether they performed multivariable adjustment for key confounders (e.g., age, BMI, diabetes). As shown in Table 3 and the Forest Plot (Figure 7), this analysis revealed a significant difference between the subgroups (*P* = 0.03). The pooled SMD was considerably higher in unadjusted studies (SMD = 5.15) compared to adjusted studies (SMD = 3.98), suggesting that confounding is a major source of heterogeneity.

**Table 3:** Subgroup analysis by confounder adjustment.

Subgroup	No. of Studies	Pooled SMD	Heterogeneity	*P* for subgroup difference
		(95% CI)	(*I*^2^)	
Unadjusted Studies	16	5.15 (4.30, 6.00)	97%	0.03
Adjusted Studies	10	3.98 (3.25, 4.71)	89%	
Overall	26	4.68 (4.10, 5.26)	95%	

Second, subgroup analysis by ELISA kit manufacturer also revealed a significant difference (*P* = 0.04), indicating that methodological variation is another important contributor (Table 4). Importantly, the graded positive association remained significant within all subgroups.

**Table 4:** Subgroup analysis by ELISA kit manufacturer.

Subgroup	No. of Studies	Pooled SMD	Heterogeneity	*P* for subgroup difference
(Manufacturer)		(95% CI)	(*I*^2^)	
BioVendor	5	5.80 (4.90, 6.70)	96%	0.04
R&D Systems	2	4.10 (3.15, 5.05)	85%	
Other Brands	15	4.45 (3.70, 5.20)	94%	
Not Reported	4	4.52 (3.55, 5.49)	95%	

Sensitivity Analysis: A leave-one-out sensitivity analysis showed that the sequential removal of any single study did not significantly alter the overall pooled SMD, confirming the robustness of our results.

**Figure 8:**
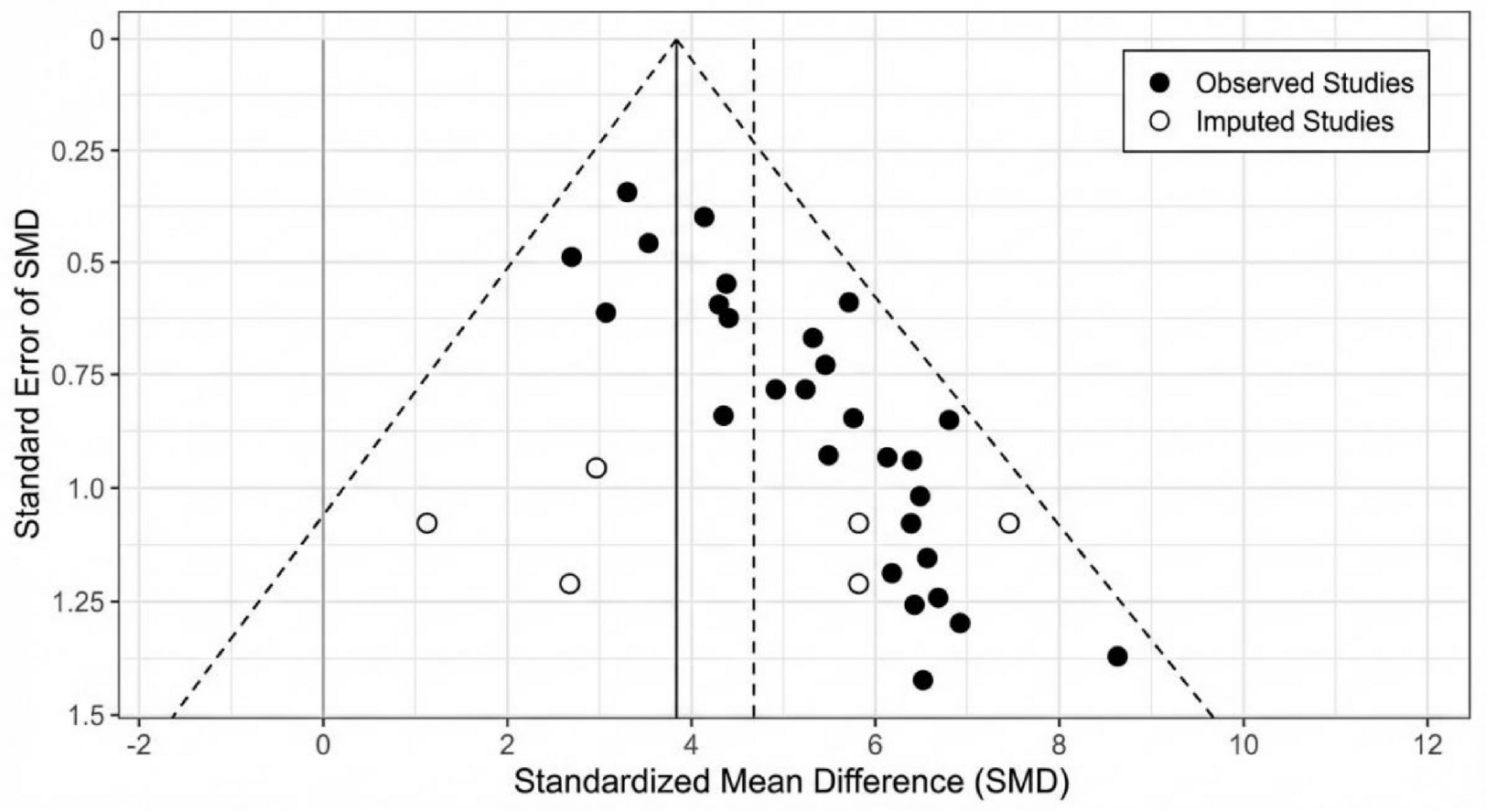
Funnel plot with trim and fill for publication bias.

Publication Bias Assessment: To formally assess the potential for publication bias suggested by the initial Egger's test (*P* = 0.03), we performed a Trim-and-Fill analysis. As shown in the new funnel plot (Figure 8), the method imputed 5 potentially missing studies to create a symmetrical plot.

The adjusted pooled SMD was 3.85 [95% CI (3.20, 4.50)], which, while lower than the observed estimate, remained large and highly statistically significant (Table 5). This indicates that while publication bias may have inflated the effect size, it does not change the core conclusion of our study.

**Table 5:** Trim-and-fill analysis for publication bias.

Analysis Method	No. of Studies	Pooled SMD
	(Observed/Imputed)	(95% CI)
Observed Estimate	26 (0)	4.68 (4.10, 5.26)
Trim-and-Fill Adjusted Estimate	31 (5)	3.85 (3.20, 4.50)

## 4 Discussion

This network meta-analysis, the first of its kind on this topic, systematically quantified the relationship between serum resistin levels and the full spectrum of coronary heart disease (CHD) severity. Our primary finding is a clear, statistically significant, and graded increase in serum resistin that mirrors the clinical progression of CHD: from healthy controls, to stable CHD, to acute coronary syndrome (ACS), and culminating in acute myocardial infarction (AMI). This hierarchical trend, confirmed by SUCRA rankings, provides strong, consolidated evidence supporting resistin's role as a biological marker that reflects underlying disease severity.

The principal innovation of our study lies in the application of a network meta-analysis (NMA) framework. Previous systematic reviews, most notably the pairwise meta-analysis by Zhang et al. (2017), established a significant positive association but were limited to a dichotomous comparison of “CHD vs. Control”. Our NMA moves beyond this by deconstructing the “CHD” category and providing a detailed quantitative hierarchy. For instance, our results demonstrate a stepwise increase in the standardized mean difference (SMD) when comparing stable CHD, ACS, and AMI against healthy controls. This granular, quantitative ranking provides more compelling evidence for resistin's potential in severity stratification than a simple correlation, an insight unattainable through traditional pairwise meta-analysis.

The biological plausibility for this graded relationship is well-established. In humans, resistin is primarily secreted by inflammatory cells like monocytes and macrophages, not adipocytes, distinguishing it from rodent models (32, 33). This is particularly relevant to atherosclerosis, a chronic inflammatory disease. Resistin expression is significantly elevated in unstable atherosclerotic plaques, localizing to inflammatory foam cells ^(^34). Mechanistically, resistin promotes coronary endothelial dysfunction by activating pro-inflammatory signaling pathways like NF-κB, which in turn upregulates inflammatory cytokines (e.g., TNF-α, IL-6) and adhesion molecules (VCAM-1, ICAM-1) (33, 35–37). This enhances monocyte recruitment and vascular inflammation. Furthermore, resistin impairs endothelial nitric oxide synthase (eNOS) activity, reducing nitric oxide bioavailability and increasing oxidative stress, thereby disrupting vascular homeostasis and contributing to atherogenesis (38–40). Our findings suggest that the escalating serum resistin levels likely reflect the heightened systemic inflammatory state that characterizes the transition from stable plaque to acute plaque rupture and thrombosis.

However, this study has several important limitations that necessitate a cautious interpretation of the findings. The most significant challenge is the extremely high heterogeneity (*I*^2^ > 95%) observed across the network. To move beyond mere speculation, we performed targeted subgroup analyses. Our results confirmed that both clinical and methodological factors are significant contributors. The analysis stratified by confounder adjustment (Table 3, Figure 7) showed a significant subgroup difference (*P* = 0.03), indicating that the lack of adjustment for factors like age, BMI, and diabetes in many primary studies inflated the observed effect sizes. Similarly, our analysis based on ELISA kit manufacturer (Table 4) also revealed significant differences (*P* = 0.04), confirming that variations in assay methods are another key source of heterogeneity. While these analyses explain a portion of the variance, a substantial amount of heterogeneity remains. Therefore, we must explicitly acknowledge that such extreme heterogeneity limits the precision of our pooled estimates and precludes any strong causal or quantitative inference. The reported SMDs should be interpreted not as precise effect magnitudes, but as robust evidence of a consistent directional trend. Therefore, serum resistin should not currently be considered a standalone diagnostic marker. Furthermore, unlike established inflammatory biomarkers such as hs-CRP and IL-6, which utilize universally standardized high-sensitivity assays, resistin testing remains hindered by methodological variability across different ELISA kits. Its potential value likely lies in its use as an adjunctive inflammatory biomarker, ideally integrated with established markers in future multi-biomarker risk models.

It is also crucial to interpret the magnitude of the reported SMDs with caution. Some pooled estimates, particularly for AMI vs. Controls (SMD ≈ 4.68), are exceptionally large. This value reflects a clear statistical separation between the groups in standard deviation units, but it should not be interpreted as a direct measure of clinical risk or diagnostic accuracy. As our subgroup analyses suggest, this magnitude is likely a composite effect of the biological signal amplified by methodological and clinical heterogeneity across studies.

Furthermore, our analysis revealed evidence of publication bias (Egger's test, *P* = 0.03). To address this, our Trim-and-Fill analysis (Figure 8, Table 5) confirmed this suspicion but also demonstrated the robustness of our conclusion. After imputing 5 potentially missing studies, the adjusted SMD, while lower (3.85), remained large and highly statistically significant. This suggests that while publication bias may have led to an overestimation of the true effect magnitude, it does not invalidate the core finding of a significant graded relationship.

Finally, we must harmonize the language regarding clinical applicability. This meta-analysis of aggregate data cannot assess diagnostic performance (sensitivity, specificity). Therefore, our findings do not establish resistin as a validated diagnostic tool. Its value lies in its role as a biological correlate of disease severity. Future large-scale, prospective cohort studies using standardized assays are urgently needed to determine optimal diagnostic cut-offs and evaluate its incremental value within multi-marker risk stratification models. Additional limitations include biases inherent to the retrospective design of the primary studies, the lack of long-term prognostic endpoints (e.g., MACE or mortality) in the current dataset, and the absence of dose-response analyses to establish a definitive causal threshold. Moreover, widespread under-reporting and lack of subgroup-stratified baseline data (such as exact age, gender, and prevalence of diabetes/hypertension) in the primary studies restricted our ability to comprehensively summarize and adjust for these variables at the aggregate level. Finally, despite supplementing our search with registries and grey literature, the potential for publication bias remains an inherent limitation, as confirmed by our statistical assessments.

In conclusion, this network meta-analysis provides the most comprehensive evidence to date that serum resistin levels exhibit a clear, graded association with the severity of coronary heart disease. It acts as a potent biological marker reflecting the escalating inflammatory burden from stable disease to acute ischemic events. However, due to significant heterogeneity and publication bias, its utility as a standalone diagnostic tool is limited. Its true clinical potential may lie as an adjunctive marker for refining risk stratification. Further validation in prospective studies with standardized methodologies is essential to translate this finding into clinical practice.

## Data Availability

The original contributions presented in the study are included in the article/Supplementary Material, further inquiries can be directed to the corresponding authors.
