# Supplementary material for: Serum resistin as a potential biomarker for stratifying the severity of coronary heart disease: a network meta-analysis
Source: Front Cardiovasc Med. 2026 May 28;13:1774421. doi: 10.3389/fcvm.2026.1774421 (PMC13253230; doi:10.3389/fcvm.2026.1774421)
Supplement: Supplementary file 1 [file Datasheet1.pdf]

Supplementary Table S2

Comparison	Number of Studies (k)	SMD (95% CI)	P value	Heterogeneity (I^2)	τ^2
Stable CHD vs. CHD-	19	0.69 (0.41, 0.98)	<0.001	95.7%	0.333
ACS vs. CHD-	7	1.40 (0.36, 2.44)	0.008	97.7%	1.886
AMI vs. CHD-	7	5.74 (3.25, 8.23)	<0.001	98.5%	10.835
ACS vs. Stable CHD	7	1.88 (1.08, 2.67)	<0.001	97.7%	1.051
AMI vs. Stable CHD	6	1.54 (0.71, 2.36)	<0.001	94.7%	0.927
AMI vs. ACS	3	1.05 (-0.15, 2.26)	0.087	95.5%	1.048

Supplementary Table S4: Node-splitting analysis of local inconsistency

Comparison	Direct Estimate (SMD, 95% CI)	Indirect Estimate (SMD, 95% CI)	Difference (95% CI)	P-value
Stable CHD vs. CHD-	0.69 (0.41, 0.98)	1.10 (-0.15, 2.35)	-0.41 (-1.69, 0.87)	0.529
ACS vs. CHD-	1.40 (0.36, 2.44)	2.25 (0.45, 4.05)	-0.85 (-2.92, 1.22)	0.423
AMI vs. CHD-	5.74 (3.25, 8.23)	4.35 (3.50, 5.20)	1.39 (-1.21, 3.99)	0.298
ACS vs. Stable CHD	1.88 (1.08, 2.67)	0.75 (-0.45, 1.95)	1.13 (-0.31, 2.57)	0.123
AMI vs. Stable CHD	1.54 (0.71, 2.36)	4.85 (-0.50, 10.20)*	-3.31 (-8.74, 2.12)	0.234
AMI vs. ACS	1.05 (-0.15, 2.26)	3.50 (1.25, 5.75)	-2.45 (-5.00, 0.10)	0.059

*(Note: All P-values > 0.05 indicate no significant inconsistency between direct and indirect evidence. The wide confidence interval in the indirect estimate reflects the substantial heterogeneity observed across the network loops.)
